# Cell-Free and In Vivo Characterization of the Inhibitory Activity of *Lavado* Cocoa Flavanols on the Amyloid Protein Ataxin-3: Toward New Approaches against Spinocerebellar Ataxia Type 3

**DOI:** 10.1021/acschemneuro.3c00560

**Published:** 2023-12-28

**Authors:** Barbara Sciandrone, Alessandro Palmioli, Carlotta Ciaramelli, Roberta Pensotti, Laura Colombo, Maria Elena Regonesi, Cristina Airoldi

**Affiliations:** † Department of Biotechnology and Biosciences, University of Milano-Bicocca, P.zza Della Scienza 2, 20126 Milan, Italy; ‡ NeuroMI, Milan Center for Neuroscience, University of Milano-Bicocca, 20126 Milano, Italy; § Department of Molecular Biochemistry and Pharmacology, Istituto di Ricerche Farmacologiche Mario Negri IRCCS, Via M. Negri 2, 20156 Milano, Italy

**Keywords:** spinocerebellar ataxia type 3 (SCA3), ataxin-3 protein (ATX3), flavanols, *Lavado* cocoa, NMR, UPLC-HR-MS, *Caenorhabditis elegans*

## Abstract

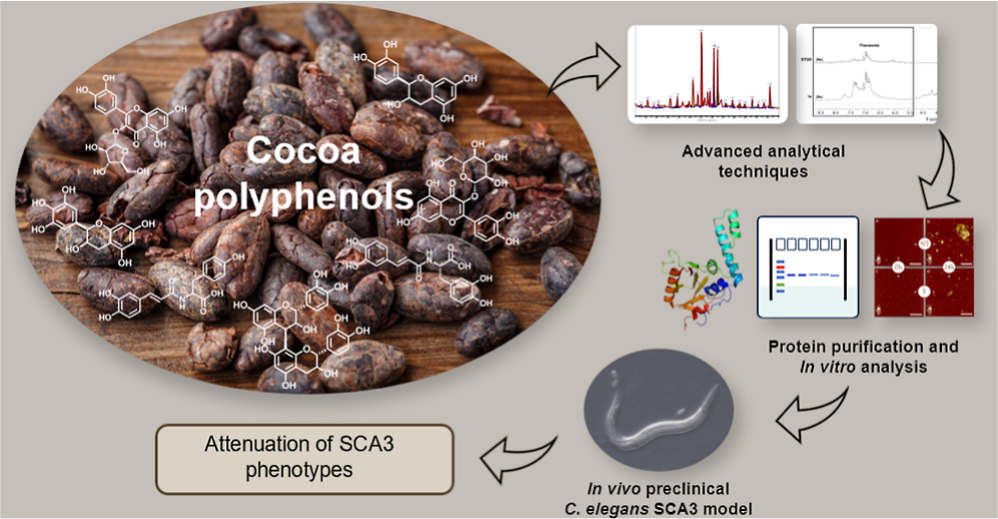

Spinocerebellar ataxia type 3 (SCA3) is a neurodegenerative disorder characterized by ataxia and other neurological manifestations, with a poor prognosis and a lack of effective therapies. The amyloid aggregation of the ataxin-3 protein is a hallmark of SCA3 and one of the main biochemical events prompting its onset, making it a prominent target for the development of preventive and therapeutic interventions. Here, we tested the efficacy of an aqueous *Lavado* cocoa extract and its polyphenolic components against ataxin-3 aggregation and neurotoxicity. The combination of biochemical assays and atomic force microscopy morphological analysis provided clear evidence of cocoa flavanols’ ability to hinder ATX3 amyloid aggregation through direct physical interaction, as assessed by NMR spectroscopy. The chemical identity of the flavanols was investigated by ultraperformance liquid chromatography-high-resolution mass spectrometry. The use of the preclinical model *Caenorhabditis elegans* allowed us to demonstrate cocoa flavanols’ ability to ameliorate ataxic phenotypes in vivo. To the best of our knowledge, *Lavado* cocoa is the first natural source whose extract is able to directly interfere with ATX3 aggregation, leading to the formation of off-pathway species.

## 1 Introduction

Spinocerebellar ataxia type 3 (SCA3), also known as Machado-Joseph disease, is the most common subtype of autosomal dominant cerebellar ataxia, a neurodegenerative disorder characterized by ataxia, external progressive ophthalmoplegia, and other neurological manifestations.^1,2^ The disease belongs to the polyglutamine (polyQ) group and is associated with a CAG repeat expansion mutation in the polyQ tract of the ATXN3 gene (14q21) with the anticipation phenomenon.^3^ The gene encodes for the ataxin-3 protein (ATX3), in which the normal glutamine repeat number is 13–41 residues, whereas the polyQ tract length causing SCA3 is greater than 55.^1,2^ The mutation leads to the formation of toxic aggregates of ATX3 in some brain cell types, which ultimately lead to cell death.^4^ ATX3 consists of a globular N-terminal domain, the so-called Josephin domain (JD), that triggers the aggregation process of the full-length protein and displays amyloidogenic properties when incubated alone.^5^ The C-terminal flexible tail^6,7^ contains the polyQ tract that induces the formation of the sodium dodecyl sulfate (SDS)-insoluble fibers only in the expanded ATX3 variant.^8−10^ Much effort is being devoted to the development of therapeutic strategies capable of contrasting this process, thus preventing, or at least retarding, neurodegeneration.^11^ However, no effective therapeutic intervention is currently available. In previous works, we assayed the binding and the effect of the polyphenol epigallocatechin-3-gallate (EGCG) on the aggregation pattern and toxicity of an expanded pathogenic ATX3 variant (ATX3Q55).^12−14^ This compound mitigated the protein’s toxic effects, as shown in the*Caenorhabditis elegans*model, by expressing the expanded protein in the nervous system. At the same time, we also observed that, when incubated in the presence of EGCG, ATX3 aggregation gave rise to off-pathway, soluble, SDS-resistant, nontoxic species.^12,14^

Recently, our group reported the neuroprotective potential of the cocoa extract to counteract Aβ1–42 peptide aggregation and neurotoxicity. Flavanols, in particular catechins and their derivatives, were demonstrated to be cocoa components mainly responsible for these inhibitory activities.^15^ This observation prompted us to investigate the potential anti-ATX3 activity of our extracts, in particular *Lavado* cocoa, which had proved to be the most effective in our previous study on Aβ. To this end, we also performed a more in-depth chemical analysis of *Lavado* cocoa extract and its polyphenolic-enriched fraction, previously characterized only by NMR spectroscopy,^15^ by ultraperformance liquid chromatography (UPLC)-high-resolution mass spectrometry (HR-MS). Then, the *Lavado* cocoa total extract and, in particular, its polyphenol-enriched fraction were assessed for their ability to prevent ATX3 aggregation in a cell-free system and to reduce toxicity in the ataxic *C. elegans* model.

Collectively, our data show cocoa flavanols’ ability to contrast ATX3 amyloid aggregation through direct physical interaction and to ameliorate ataxic phenotypes in vivo.

## 2 Results and Discussion

### 2.1 *Lavado* Cocoa Extract and Polyphenol-Enriched Fraction Characterization

**Figure 1:**
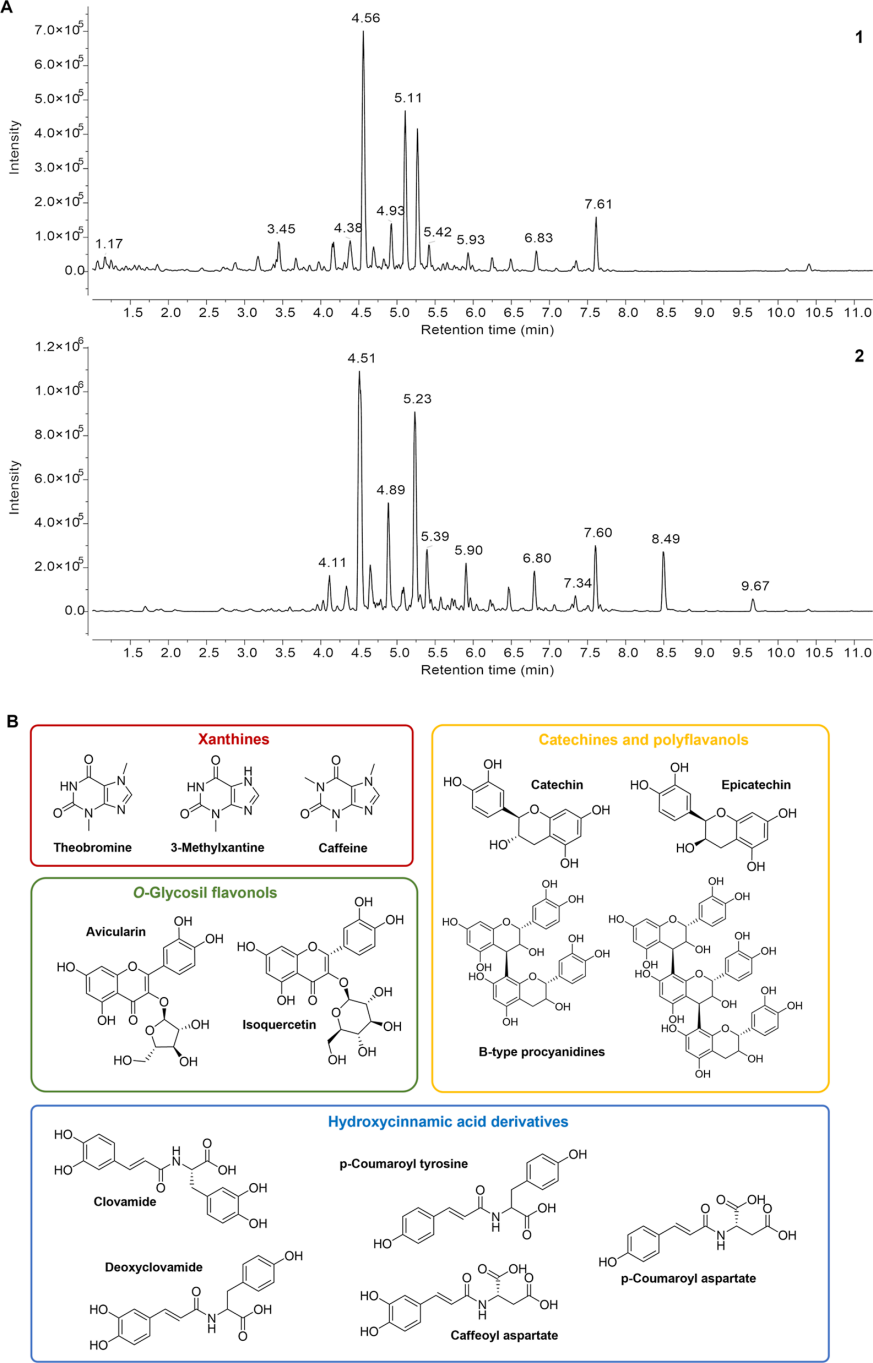
UPLC-HR-MS analysis of *Lavado* cocoa extracts. Base peak chromatograms obtained under the negative ionization mode (A) of total extract (1) and its related polyphenol-enriched fraction (2). Structures of major compounds identified in *Lavado* cocoa extract and its related polyphenol-enriched fraction (B).

The NMR-based metabolic profiling of *Lavado* cocoa and its polyphenol-enriched fraction was already reported by our group.^15^ Here, their chemical composition, in terms of aromatic and, in particular, polyphenolic components, was further investigated by UPLC coupled with HR-MS, also monitoring the separation through a photodiode array (PDA) detector to reveal the characteristic polyphenol absorbances at 280 and 320 nm. Results are summarized in Figure 1, reporting the base peak chromatograms under negative ionization (A) and the structures of major identified compounds (B), and in Supporting Information—Table S1, containing spectrometric data. We identified a total of 21 aromatic compounds, belonging to four main classes: catechins and poly flavanols (catechin, epicatechin, and B-type procyanidin dimers and trimers), *O*-glycosyl flavonoids (such as aviscularin and isoquercetin), hydroxycinnamic acid derivatives (clovamide, deoxyclovamide, caffeoyl aspartate, *p*-coumaroyl aspartate, and *p*-coumaroyl tyrosine), and xanthines (3-methyl xanthine, theobromine, and caffeine) (Figure 1 and Supporting Information—Table S1). Four metabolites remained unknown (Table S1).

Notably, the comparison of chromatograms 1 (total extract) and 2 (polyphenol-enriched fraction) clearly revealed that polyphenols present in the total extract (1) were all retained in the enriched fraction (2), as indicated by our preliminary NMR analysis, which clearly showed the enrichment in polyphenol components,^15^ but without the identification of the individual molecules.

### 2.2 *Lavado* Cocoa Extract Affects JD Aggregation Kinetics and Solubility

The antiamyloidogenic properties of the *Lavado* cocoa extract were evaluated on purified JD. The rationale of this approach relies on the well-established role of this domain in the aggregation process of full-length ATX3 that starts with the JD conformational transition from native spheroidal oligomers through the conversion of β-enriched oligomers to mature fibers. However, the addition of the unordered region and the expansion of the polyQ tract induce the fastest aggregation kinetics and the formation of oligomeric toxic species.^8−10^ Thus, quite plausibly, any treatment affecting or preventing JD aggregation would also interfere with the fibrillogenesis of full-length ATX3.

**Figure 2:**
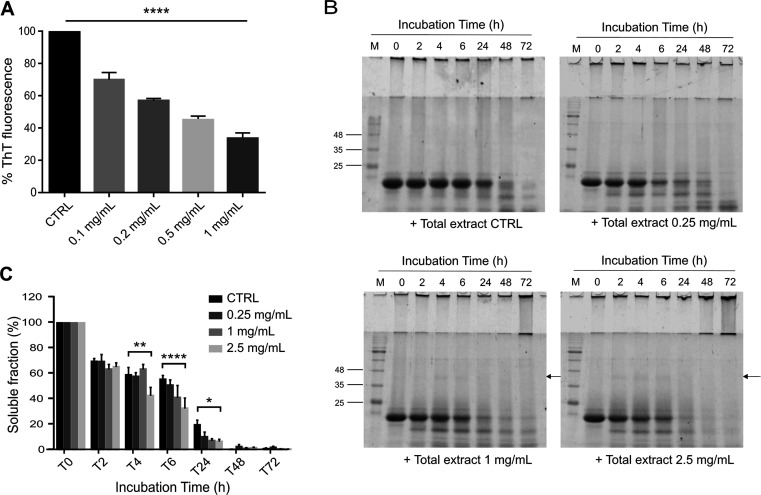
Analysis of the *Lavado* cocoa extract effect on JD aggregation in cell-free system. (A) Effect of *Lavado* cocoa extract on JD evaluated by the ThT fluorescence assay. Different concentrations of *Lavado* cocoa extract (0, 0.1, 0.2, 0.5, and 1 mg/mL) were incubated with JD (50 μM) at 37 °C, and ThT fluorescence was monitored for 24 h. The data represents the mean ± standard error of the maximum fluorescence values reached at each concentration after 24 h, subtracted from the fluorescence of the related control (ThT and cocoa extract), and normalized to untreated JD (B,C). Solubility assay of JD incubated in the presence or absence of *Lavado* cocoa extract. Aliquots of 50 μM JD were incubated in phosphate-buffered saline (PBS) at 37 °C with different concentrations of *Lavado* cocoa extract (0, 0.25, 1, and 2.5 mg/mL). The soluble fractions obtained by centrifugation at different times (0, 2, 4, 6, 24, 48, and 72 h) were subjected to SDS-polyacrylamide gel electrophoresis (PAGE) (14%), stained with EZBlue gel staining solution, and scanned at 700 nm with the Odyssey Fc System [LI-COR; (B)]. The plot represents the mean ± standard error of densitometric analysis of monomeric JD expressed as a percentage of protein amount at *t* = 0 h for each concentration (C). Significant differences were assessed by a 2-way factorial analysis of variance (2-way ANOVA), followed by Dunnett’s multiple comparisons test. Arrows indicate SDS-resistant aggregated species. All data were derived from at least three independent experiments. *P < 0.05; **P < 0.01; and ****P < 0.0001.

First, the Thioflavin T (ThT) assay was used to analyze the effect of the extract on the JD aggregation.^16,17^ ThT is considered a preliminary test useful to estimate the range of concentrations of the cocoa extract to employ in the following experiments. Freshly prepared His-tagged monomeric JD (50 μM) was incubated with ThT at 37 °C in the presence or absence of different extract concentrations (Figure 2A).

After 24 h of incubation, the addition of the extract led to a progressive decrease of the maximum fluorescent value achieved compared with untreated JD (Figure 2A). The maximum reduction of ∼70% in ThT fluorescence was obtained using 1 mg/mL cocoa extract (the highest concentration tested) (Figure 2A). This could be due to two different mechanisms: (i) the capability of the extract to inhibit and slow down the JD aggregation, and (ii) the capability of the extract to lead to the formation of off-pathway aggregates with a lower ability to bind the ThT.

To better understand how JD aggregation is influenced by cocoa extract, a solubility assay was performed by taking aliquots of freshly purified JD protein incubated with and without different extract concentrations at different incubation times. The soluble fraction obtained by centrifugation was analyzed by SDS-PAGE (Figure 2B). The densitometric analysis of the SDS-soluble fractions (identified as the proteins migrating in the gels in monomeric form) showed that the extract treatment induces a faster decrease of the amount of the SDS-soluble fractions in the untreated samples. In particular, the effect is significant from the earliest times of incubation (4 h) at the highest concentration (2.5 mg/mL) (Figure 2C). In addition, it is possible to detect in the gels a higher molecular weight band (arrow in Figure 2B) whose intensity increases at higher concentrations. These aggregates could be attributable to soluble SDS-resistant species previously observed after EGCG treatment.^12^

### 2.3 *Lavado* Cocoa Polyphenol-Enriched Fraction is Responsible for the Effect on JD Aggregation

**Figure 3:**
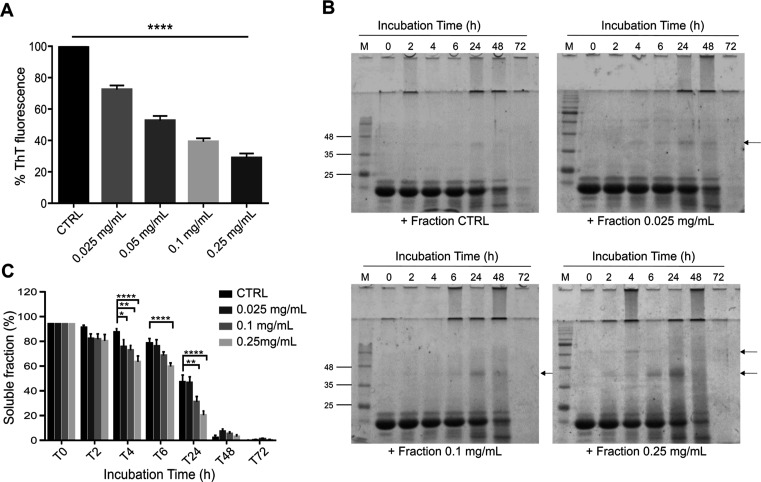
Cell-free effects of *Lavado* cocoa polyphenol-enriched fraction on JD aggregation. (A) Effect of *Lavado* cocoa polyphenol-enriched fraction on JD evaluated by a ThT fluorescence assay. JD (50 μM) protein was incubated with different concentrations (0, 0.01, 0.025, 0.05, and 0.1 mg/mL) of polyphenolic fraction at 37 °C. The ThT fluorescence was measured for 24 h and the graph represents the mean ± standard error of the maximum fluorescence values reached at each concentration, subtracted from the fluorescence of the related control (ThT and polyphenolic fraction), and normalized to JD. (B,C) Solubility assay of JD incubated with or without the polyphenol-enriched fraction. Different concentrations of PF (0, 0.025, 0.1, and 0.25 mg/mL) were incubated with 50 μM JD in PBS at 37 °C. At different times (0, 2, 4, 6, 24, 48, and 72 h), samples were centrifugated, and the soluble fractions were subjected to SDS-PAGE (14%), stained with EZBlue gel staining solution, and scanned at 700 nm with the Odyssey Fc System (LI-COR; B). Bars represent the mean ± standard error of densitometric analysis of monomeric JD expressed as a percentage of protein amount at *t* = 0 h for each concentration (C). Significant differences were assessed by a 2-way factorial analysis of variance (2-way ANOVA), followed by Dunnett’s multiple comparisons test. Arrows indicate SDS-resistant aggregated species. All data were derived from at least three independent experiments. *P < 0.05; **P < 0.01; ****P < 0.0001.

To assess whether the activity observed on JD aggregation was due to the polyphenols present in the extract, ThT and solubility test assays were replicated on JD using a *Lavado* cocoa polyphenol-enriched fraction obtained as previously reported,^15^ whose chemical composition was determined (Figure 1 and Table S1). Freshly prepared JD was incubated with different concentrations of polyphenol-enriched fraction, and a significant decrease of ThT fluorescence was observed up to a maximum reduction of about 70% with the highest concentration tested (0.25 mg/mL) (Figure 3A).

Noteworthy, the polyphenol fraction showed activity on JD comparable to that displayed by the *Lavado* cocoa extract but at a concentration about 4 times lower (0.25 mg/mL for the fraction vs 1.0 mg/mL for the total extract) (Figures 2A, 3A). Furthermore, the solubility assay showed a significant decrease in monomeric JD amount since the fourth h of incubation with all the fraction concentrations tested (0.025, 0.1, and 0.25 mg/mL) (Figure 3B,C), and in the gels appeared a similar SDS-resistant band observed with the cacao total extract (Figures 2 and 3B). All of the results provide evidence that the polyphenol-enriched fraction of *Lavado* cocoa is the component mainly involved in the inhibitory activity displayed by the extract on JD aggregation.

### 2.4 *Lavado* Cocoa Polyphenol Enriched Fraction has Antiamyloidogenic Properties on ATX3Q55

**Figure 4:**
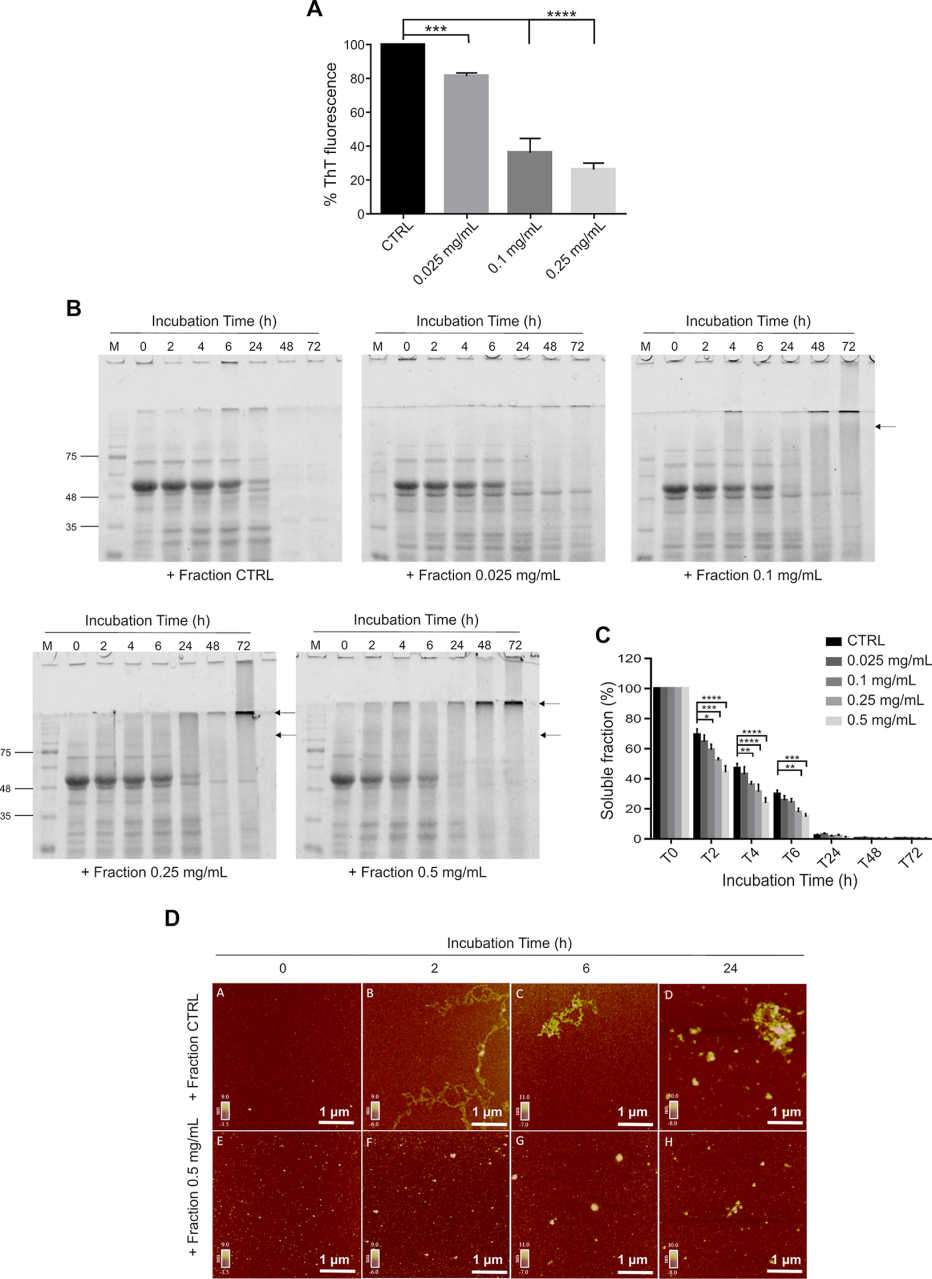
Cell-free effects of the *Lavado* cocoa polyphenolic-enriched fraction on ATX3Q55 aggregation. (A) Effect of the polyphenol-enriched fraction on ATX3Q55 evaluated by a ThT fluorescence assay. Different concentrations of polyphenol-enriched fraction (0, 0.025, 0.1, and 0.25 mg/mL) were incubated with ATX3Q55 (25 μM) at 37 °C, and ThT fluorescence was measured for 24 h. The means ± standard error of the maximum fluorescence values reached for each concentration at 24 h subtracted from the fluorescence value of the related control (ThT and polyphenolic fraction) are normalized to ATX3Q55. (B,C) Solubility assay of ATX3Q55 incubated in the presence of a polyphenol-enriched fraction. Aliquots of 25 μM ATX3Q55 were incubated in PBS with different concentrations of polyphenol-enriched fraction (0, 0.025, 0.1, 0.25, and 0.5 mg/mL) at 37 °C. After centrifugation at different time points (0, 2, 4, 6, 24, 48, and 72 h), the soluble fractions were obtained and analyzed by SDS-PAGE (12%). Gels were stained with EZBlue gel staining solution and scanned at 700 nm with an Odyssey Fc System (LI-COR; B). The densitometric analysis of monomeric ATX3Q55 protein was plotted and represents the mean ± standard error as a percentage of the protein amount at *t* = 0 h for each concentration (C). For the statistical analysis, significant differences were assessed by a 2-way factorial analysis of variance (2-way ANOVA), followed by Dunnett’s multiple comparisons test. Arrows indicate SDS-resistant aggregated species. All data were derived from at least three independent experiments. *P < 0.05; **P < 0.01; ***P < 0.001; ****P < 0.0001. (D) Atomic force microscopy (AFM) morphological analysis of the ATX3Q55 aggregates. AFM images were acquired on a sample containing 25 μM ATX3Q55 incubated in the absence (1–4) or the presence (5–8) of the polyphenol-enriched fraction (0.5 mg/mL) for different times (0, 2, 4, 6, and 24 h).

To further validate the antiamyloidogenic properties of the polyphenol-enriched fraction, ThT and solubility assays were performed on the ATX3 expanded protein, carrying 55 Gln in the polyQ tract (ATX3Q55). After 24 h of coincubation, the polyphenol-enriched fraction results in effective inhibition of amyloidogenic protein aggregation at all concentrations, with a reduction from 20% (with 0.025 mg/mL) up to 70% (with 0.25 mg/mL) of ThT fluorescence (Figure 4A).

The analysis of soluble fractions in SDS-PAGE clearly confirmed the ability of the polyphenol-enriched fraction to accelerate the aggregation process of AT3Q55, which was significant even at the lowest concentrations and from the first 2 h of incubation (Figure 4B,C). Moreover, fraction addition induces the formation of soluble SDS-resistant species of high molecular weight at the early times of incubation (some ATX3Q55 aggregates do not get into the gels after SDS and boiling treatment; Figure 4B). The bands underlying the monomeric form of ATX3Q55 that appeared during incubation are degraded forms of the protein due to the autoproteolytic activity of ATX3, as previously demonstrated.^18^ However, protease inhibitors were not added to the samples because they accelerated the ATX3Q55 aggregation process (data not shown).

To further understand the mechanism of action of the cocoa polyphenols, tapping mode AFM was performed to analyze the morphology of the aggregates formed during AT3Q55 aggregation in the presence of the polyphenol-enriched fraction (Figure 4D). To this aim, ATX3Q55 protein was incubated alone (1–4) or with 0.5 mg/mL of the polyphenol-enriched fraction (5–8) at 37 °C,

and samples were collected after 0, 2, 6, and 24 h of incubation. AFM images clearly show the aggregation of the untreated protein, with the formation of fibrillar structures as early as 2 h (2), which then evolve to give large clusters, i.e., amyloid mature fibers, at 24 h (4). Conversely, the presence of the polyphenolic fraction prevents the formation of fibrillar aggregates, while we witness the appearance of only small amorphous aggregates (6–8).

Taken together, the results on ATX3Q55 clearly demonstrate the antiamyloidogenic properties of *Lavado* cocoa polyphenols, i.e., their capability to accelerate ATX3Q55 aggregation and to lead to the formation of SDS-resistant aggregates of reduced size.

### 2.5 Cocoa Flavanols Directly Interact with ATX3

**Figure 5:**
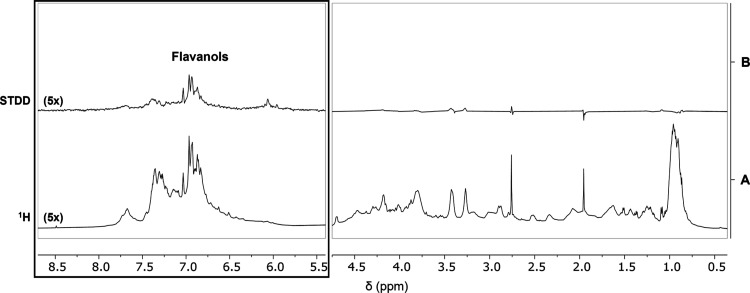
STD NMR characterization of *Lavado* cocoa polyphenols’ interaction with ATX3Q55 protein. ^1^H NMR (A) and STDD (B) spectra of a mixture of *Lavado* cocoa polyphenol-enriched fraction (10 mg/mL) and ATX3Q55 protein (7 μM) in PBS (10% D_2_O), pH 7.2. STD spectra were acquired with 1024 scans and a 2 s saturation time at 600 MHz, 25 °C. An amplification factor of 5 (5×) of the aromatic regions was used to optimize the visualization of the signals of interest in each spectrum.

STD NMR^19,20^ is a powerful tool to study molecular interactions involving amyloid proteins, as demonstrated by our group for ATX3Q55 proteins^12,14,21^ and Aβ peptides.^22−25^ In addition, this technique allows the easy and rapid screening of complex mixtures of molecules, such as natural extracts, for the presence of amyloid oligomer ligands.^13,26−30^ With the same approach used to find Aβ oligomers in cocoa,^15^ the polyphenol-enriched fraction of *Lavado* cocoa was submitted to STD NMR studies to identify the components capable of interacting directly with ATX3Q55 protein. A sample containing a mixture of *Lavado* cocoa polyphenol-enriched fraction (10 mg/mL) and ATX3Q55 protein (7 μM) dissolved in PBS, pH 7.2, was prepared to afford the corresponding STD experiments. Under these experimental conditions, as also assessed by AFM analysis (Figure 4E), the protein is mainly in the oligomeric state. The ^1^H spectrum is depicted in Figure 5A. A blank STD reference experiment was acquired under the same experimental conditions on the polyphenol fraction alone. The saturation transfer double difference (STDD) NMR spectrum^15,31,32^(Figure 5B) was achieved by subtracting the blank STD spectrum from the STD spectrum acquired in the presence of the protein.

The STDD spectrum contains resonances belonging mainly to flavanols, already identified by UPLC-HR-MS (Figure 1 and Supporting Information, Table S1) as the main representative components of the *Lavado* cocoa polyphenol-enriched fraction. This finding supports our driving hypothesis that catechins and their polymers (procyanidins), particularly abundant in cocoa, may possess antiamyloidogenic activity also against ATX3 (as well as assessed for Aβ1–42).^15^

### 2.6 Polyphenol-Enriched Fraction Ameliorates the Health Span of the *C. elegans* SCA3 Model

The antiamyloidogenic activity exerted by the *Lavado* cocoa polyphenol-enriched fraction was also tested in vivo using a SCA3 model of *C. elegans* expressing a pathological ATX3 protein carrying 130 glutamines (ATX3Q130) only in neuronal cells. As a control, we used a *C. elegans* strain expressing a healthy protein with 17 glutamines (ATX3Q17).^11^ This allows us to discriminate between the antiamyloidogenic properties of *Lavado* cocoa polyphenol-enriched fraction and the negative effect produced by the heterologous protein overexpression. 0.5 mg/mL of the polyphenol-enriched fraction was daily administered from the first day of adulthood with killed OP50 as a food source. Both lifespan and locomotion were scored every day until all of the worms died.

**Figure 6:**
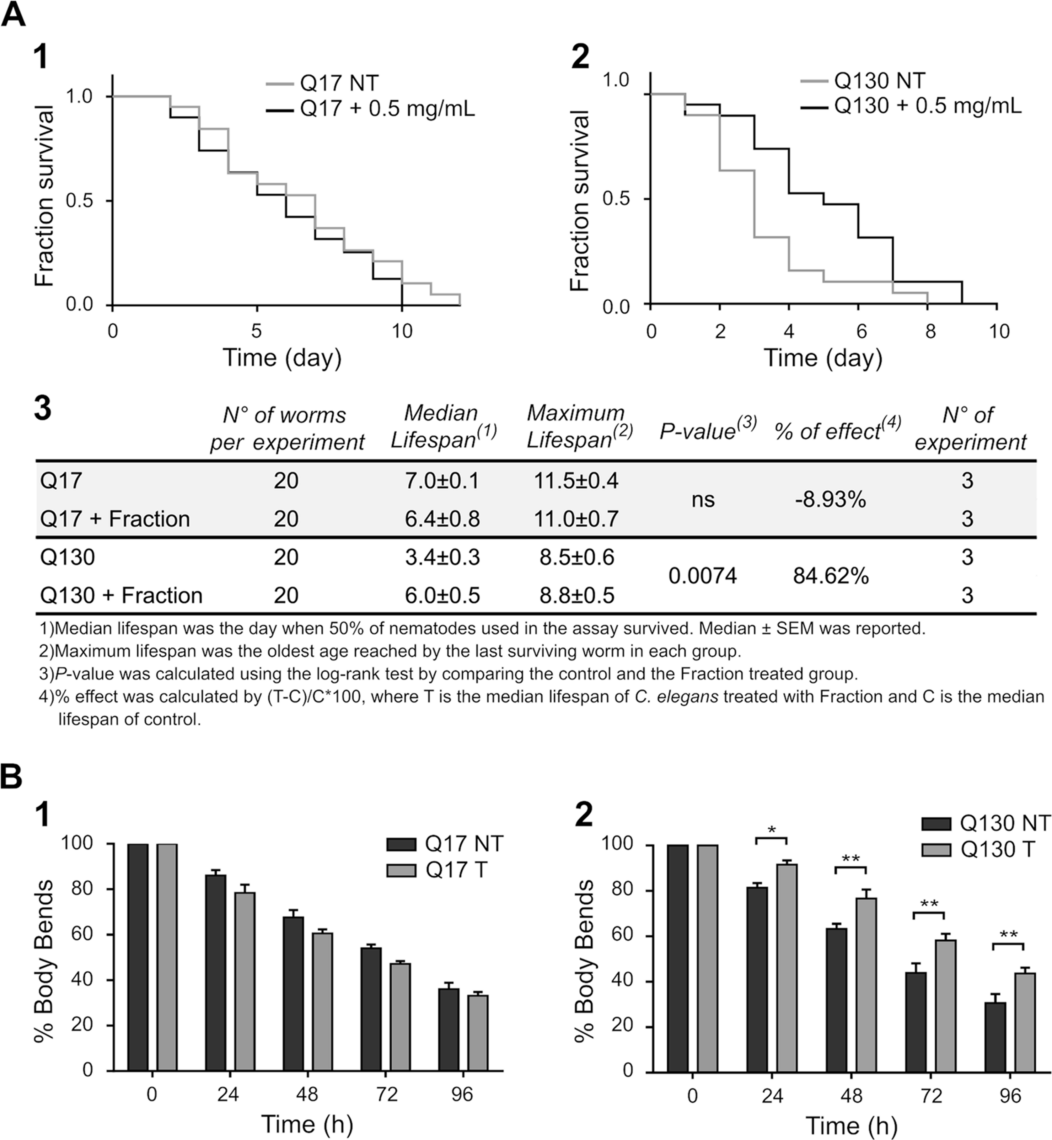
Polyphenol fraction effect on ATX3 transgenic *C. elegans* lifespan and motility. (A1-2-3) Effect of *Lavado* cocoa polyphenol-enriched fraction on *wild-type* and ataxic *C. elegans* strains survival. One-day synchronized adult worms were placed on a plate seeded with heat-killed OP50 in the presence or absence of 0.5 mg/mL of a polyphenol-enriched fraction at 25 °C. Nematodes were transferred daily on a new plate, and the number of alive was reported until all were dead. Representative Kaplan–Meier survival curves of AT3Q17-GFP (1) and AT3Q130-GFP (2) treated and not treated animals were reported. A statistical analysis was reported in panel 3. (B1-2) Effect of the polyphenol-enriched fraction on *wild-type* (1) and ataxic *C. elegans* strains (2) motility. One-day synchronized adult worms were placed on a plate seeded with heat-killed OP50 in the presence or absence of 0.5 mg/mL of the polyphenol-enriched fraction at 25 °C. Worms were transferred daily on a new plate, and body bends were counted for 20 s after 24, 48, 72, and 96 h of treatment. Data are expressed as the percentage of motility increase at time 0 h. Error bars represent standard errors. Each test was repeated at least four times, and for each treatment, at least 30 worms were used. Significant differences were assessed by a 2-way factorial analysis of variance (2-way ANOVA), followed by Bonferroni’s multiple comparisons test *P < 0.05; **P < 0.01; ****P < 0.0001; and #*P* < 0.00001.

The treatment did not affect the ATXQ17 lifespan (Figure 6A1). In fact, both the treated and untreated nematodes showed a maximum lifespan of about 11 days and a median lifespan of about 7 days with no significant differences (Figure 6A1). Otherwise, on the ATXQ130 strain, the treatment induced an increase in the median lifespan, i.e., from 3 days (untreated) to 6 days (treated), without extending the maximum lifespan (Figure 6A3).

Since ataxia, consisting of a lack of voluntary coordination of muscle movements, is a hallmark symptom of SCA3 disease, the body bend test was used to assess worm locomotion. Animals were treated in the same conditions used for the lifespan assay, and the body bands of each animal were counted every day for 1 min from the first day of adulthood (time 0 h) until death. For both strains, the number of body bends was reported as a percentage and normalized at time 0 h. The AT3Q17 strain treated displayed no significant difference in locomotion after treatment (Figure 6B1). Otherwise, the treatment with the polyphenol-enriched fraction on ATX3Q130 displayed a significant increase in the body bends number since day 1 of administration, with a relative increase that rose from ∼5% at 24 h to ∼25% at 96 h (Figure 6B2).

On these bases, we can argue that cocoa polyphenols exert an antiamyloidogenic activity on ATX3 fibrillation both in cell-free and in vivo systems.

## 3 Methods

### 3.1 Preparation of *Lavado* Cocoa Extracts and the Polyphenol-Enriched Fraction

Samples’ preparation was performed as already described in Ciaramelli et al. 2021.^15^ Briefly, the cocoa powder (5 g) was grounded and then treated with dichloromethane in a Soxhlet apparatus to remove the lipid fraction. The solid was dried, resuspended in Milli-Q water, and extracted once again in a Soxhlet apparatus at a water boiling point. After lyophilization, the extract was weighed and stored at −20 °C. A polyphenol-enriched fraction was obtained by preparative reverse-phase C18 column chromatography with water and methanol as eluent solvents. Fractions were pooled in homogeneous groups and eluent removed under reduced pressure, residues were freeze-dried and stored at −20 °C. Stock solutions of *Lavado* cocoa extracts and related polyphenol-enriched fractions were prepared at 10 and 5 mg/mL, respectively, in 20 mM phosphate buffer pH 7.4 and used for biological assays.

### 3.2 Extract and Polyphenol-Enriched Fraction Chemical Characterization by UPLC Coupled with ESI-HR-MS Spectrometry

HRMS analysis of *Lavado* cocoa extract and related polyphenol-enriched fractions was performed using the ACQUITY UPLC H-class system equipped with a PDA detector and coupled with the Xevo G2-XS QTof Mass Spectrometer (Waters Corp., Milford, MA, USA) through an ESI source. Samples were dissolved in 90% water −10% acetonitrile at 2 mg/mL and separated on the ACQUITY Premier HSS T3 Column (100 × 2.1 mm, 1.8 μm) coupled with the VanGuard HSS T3 Guard Column. The mobile phases were MS-grade water (A) and acetonitrile (B), both containing 0.1% formic acid, and analyte elution was performed according to the following gradient: 0–1 min, 5% B; 1–11 min, 5–50% B linear gradient; 11–12, 50–90% B, 12–15 min isocratic 90% B, and then equilibrated for further 4 min at the initial conditions (5% B) before the next sample injection. The elution was performed at a flow rate of 0.4 mL/min, and the injection volume was 2 μL. The column temperature was set at 40 °C. Accurate mass data were collected under both negative and positive ionization modes through a data-dependent acquisition mode (FastDDA), in which a full-scan survey triggered MS/MS acquisition of the five most intense ions (Top 5) in the range of 50–1200 *m*/*z*. Full scan spectra were acquired at a scan time 0.2 s, and MS/MS spectra were acquired at a scan time 0.1 s. Dynamic collision energy was set to 6–9 V for 50 Da and 60–80 V for 1200 Da. The source parameters were as follows: electrospray capillary voltage −2/+3 kV, source temperature 120 °C, and desolvation temperature 350 °C. The cone and desolvation gas flows were 50 and 1000 L/h, respectively. The mass spectrometer, calibrated with 0.5 M sodium formate and leucine-enkephalin (100 pg/μL) infused at 10 μL/min and acquired every 30 s, was used as LockMass. The MassLynx software (version 4.2) was used for instrument control, data acquisition, and data processing. MS Dial software^33^ version 4.9.221218 was used for peak picking, deconvolution, and noise level setting, and identification of metabolites was performed according to their calculated accurate mass and isotopic pattern, and structures were confirmed by comparison MS/MS spectra using the metabolomics MSP spectral kit from authentic standards, public databases, and literature.^34,35^

Notably, the NMR analysis also supported the stability of the sample for at least 2.5 days (corresponding to the total acquisition time required for 1D and 2D experiments).^15^

### 3.3 JD and ATX3Q55 Protein Expression and Purification

The JD- and ATX3Q55-encoding genes were cloned in a pET21-a vector^36^ and expressed in *Escherichia coli* BL21 Tuner (DE) pLacI [*E. coli* B F-ompT hsdSB (rB- mB-) gal dcm lacY1 (DE3) pLacI (CamR); Novagen, Germany] as His-tagged proteins. The growth conditions, the times of induction, and the purification procedures are the same as reported in Bonanomi et al. 2014^12^ for ATX3Q55 and Amigoni et al. 2019^36^ for JD.

### 3.4 Thioflavine T Assay

The ThT molecule was used to monitor the aggregation process of JD and ATX3Q55 in the presence of different concentrations of both *Lavado* cocoa extract and the polyphenol-enriched fraction. ThT assay measures changes in ThT fluorescence intensity upon binding to protein aggregates enriched by beta-sheets. Freshly purified JD and ATX3Q55, at 50 and 25 μM, respectively, were incubated in PBS buffer at 37 °C in the presence of 20 μM ThT (Sigma-Aldrich, St. Louis, MO, USA). The fluorescence was measured on a clear-bottomed black ViewPlate-96 F TC (PerkinElmer, MA, USA) using a VICTOR TM X3 Multilabel Plate Reader (PerkinElmer, MA, USA). The excitation and emission bandpass filter wavelengths were 445 and 535 nm, respectively. Although the bandpass filter used for the emission is about 50 nm away from the maximum, this still falls within the emission range of the ThT/amyloid fibrils complex, which ranges from about 450–550 nm. Furthermore, this setting allows for minimizing the light scattering that occurs during the plate reading without appreciably affecting the sensitivity of the assay. Readings were carried out from the bottom of the plates with no shaking and recorded every 30 min for 24 h. 100 μL of mineral oil and a lid were used to prevent evaporation. The ThT data were expressed as the change in ThT fluorescence by subtracting the relative control and reported as a percentage of the untreated sample. At least three independent experiments were performed.

### 3.5 Solubility Assay and Analysis of the Soluble Protein Fraction

Freshly purified JD and ATX3Q55 (50 and 25 μM, respectively) were incubated at 37 °C in PBS buffer in the presence of different concentrations of *Lavado* cocoa extract or the polyphenol-enriched fraction. Aliquots at different incubation times (0, 2, 4, 6, 24, 48, and 72 h) were centrifuged at 20,000 × g for 15 min, and 20 μL of the supernatant was immediately denatured by adding Sample Buffer 5x (final concentration: 50 mM Tris–HCl pH 6.8, 0.4% SDS, 4% Glycerol, 0.141 M 2-mercaptoethanol), boiled for 10 min, and analyzed by SDS-PAGE (14 and 12% for JD and ATX3Q55, respectively). The gels were stained with EZBlue Gel Staining Reagent (Sigma-Aldrich, St. Louis, MO, USA), scanned at 700 nm with Odyssey Fc System (LI-COR Biosciences, Lincoln, NE, USA), and the densitometry analysis was performed with the Image Studio software (LI-COR Biosciences, Lincoln, NE, USA). Data were expressed as a percentage of the protein amount at time 0 h of the control, and at least three independent experiments were performed.

### 3.6 AFM Analysis

20 μL aliquots of purified ATX3Q55 (25 μM) were incubated at 37 °C in the PBS buffer in the presence or absence of a 0.5 mg/mL polyphenol-enriched fraction. At fixed aggregation times (0, 2, 6, and 48 h), the sample was withdrawn, incubated on a freshly cleaved mica substrate for 5 min, then rinsed with Milli-Q water and dried under mild vacuum. Samples were mounted onto a Multimode AFM with a NanoScope V system (Veeco/Digital Instruments, Plainview, NY) operating in the tapping mode, and measurements were made using 0.01–0.025 Ω/cm antimony-doped silicon probes (*T*: 3.5–4.5 μm, *L*: 115–135 μm, *W*: 30–40 μm, *k*: 20–80 N/m, f0: 323–380 kHz; Bruker AFM probes) with a scan rate in the 0.5–1.2 Hz range, proportional to the area scanned. Measurements confirmed all of the topographic patterns in at least four separate areas. To exclude interference from any artifacts, freshly cleaved mica DISCS soaked with 30 μL of PBS buffer were also used and analyzed as controls. Data analysis was performed with the Scanning Probe Image Processor (SPIP Version 5.1.6, released on April 13, 2011) data analysis package.

### 3.7 NMR Interaction Studies

NMR experiments were acquired on a Bruker 600 MHz AVANCE III equipped with a QCI cryoprobe. *Lavado* cocoa polyphenol-enriched fraction was dissolved in PBS, pH 7.2, at a 10 mg/mL concentration, and an aliquot of ATX3Q55 solution, dissolved in the same buffer, was added to reach the final protein concentration of 7 μM. Basic sequences were employed for ^1^H and STD NMR. The water signal was suppressed by excitation sculpting. ^1^H spectra were acquired with 256 scans and a 2 s recycle delay. For STD NMR experiments, a train of Gaussian-shaped pulses each of 50 ms was employed to saturate selectively the protein envelope; the total saturation time of the protein envelope was 2 s. STD experiments were acquired with 1024 scans and a saturation frequency of −1.00 ppm. On- and off-resonance spectra were acquired in an interleaved mode with the same number of scans. The STD NMR spectrum was obtained by subtracting the on-resonance spectrum from the off-resonance spectrum. Acquisitions were performed at 25 °C.

### 3.8 *Caenorhabditis elegans* Strains

Two ATX3 transgenic strains were previously developed in the *lin*-*15*(*n765ts*) *C. elegans* strain, as described in Bonanomi et al. 2014.^12^ Both strains were maintained at 25 °C on solid Nematode Growth Medium (NGM; 50 mM NaCl, 2.5 g/L peptone, 17 g/L agar; 1 mM CaCl_2_, 1 mM MgSO_4_, 5 μg/mL cholesterol in ethanol) and seeded with the *E. coli* OP50 strain for food, according to standard procedures.^12^

### 3.9 Worm Age-Synchronization

Ten adult worms were placed on a 3 mL NGM plate seeded with OP50 and allowed to lay eggs for 16 h at 25 °C. Then, adult worms were removed from the plates, and newly laid eggs were grown for 3 days at 25 °C. Synchronized adult worms were then transferred to a new NGM plate to perform experiments. Fluorescent ATX3Q17 and ATX3Q130 worms were selected using SteREO Discovery.V12 (Zeiss, Oberkochen, Germany).

### 3.10 Body Band Assay

30 adult one-day-synchronized worms were placed on a new plate in the presence or absence of 0.5 mg/mL of polyphenol-enriched fraction and heat-killed OP50 to avoid the possibility that the treatments with the fraction could directly affect *E. coli* and thus indirectly the nematodes.^37^ Body bends per minute were counted under a microscope (Leica MZ FLIII, Leica Microsystem), as described before.^36^ Body bands/min were scored every 24 h for 5 days of treatment after moving nematodes in a new plate. Data were processed and presented as a percentage of motility increase using (T – NT)/NT × 100, where T = mean body bends/min of *C. elegans* treated with polyphenol-enriched fractions and NT = mean body bends number/min of nontreated relative strain. Each test was repeated at least three times.

### 3.11 Lifespan Assay

Adult worms (*N* = 30) synchronized, as described above, were transferred onto fresh NGM plates seeded with heat-killed *E. coli* OP50 in the presence or absence of 0.5 mg/mL of polyphenol-enriched fraction. Animals were counted and transferred every day until all the nematodes were dead. Survival curve and statistical analysis were performed with GraphPad Prism 6; *p*-values were obtained using the log-rank test. At least three independent experiments were performed.

## 4 Conclusions

SCA3 is a deadly neurodegenerative disease against which neither cures, as the neuronal damage produced is irreversible, nor therapeutic strategies that delay its progression are available.^11,38^ Since the amyloid aggregation of the ATX3 protein plays a pivotal role in the disease insurgence, targeting this process through the administration of substances able to prevent or block this event represents a promising therapeutic strategy.^39−41^

Furthermore, being that SCA3 is an easily diagnosable genetic disease, substances possessing such activity could also be used for prophylactic purposes, preventing the onset of the pathology in predisposed subjects.

EGCG is among the very few compounds endowed with anti-ATX3 activity in vitro and in the model organism *C. elegans*. Cocoa is among the natural sources richest in catechins, the class of flavonoids to which EGCG belongs. *Lavado* cocoa extracts, and in particular their polyphenolic fraction, showed potent antiamyloidogenic activity against the Aβ1–42 peptide, a hallmark of Alzheimer’s disease.^15^ For these reasons, we tested the efficacy of our cocoa extracts against ATX3 protein aggregation and toxicity.

Our experimental data clearly show that *Lavado* cocoa total extract hinders JD aggregation along the amyloidogenic pathway. The inhibitory activity increases about four times when the protein is treated with the extract fraction enriched in polyphenols, capable of blocking the formation of amyloid fibers on the expanded protein ATX3Q55, as assessed by the ThT fluorescence assay, the SDS-PAGE protein solubility assay, and the AFM morphological analysis.

The capability of interfering with the aggregation of both JD and ATX3Q55 clearly suggests that cocoa components target both ATX3 monomers and oligomers. Moreover, they induce off-pathway aggregation of these proteins. This strongly correlates with the mechanism of action previously observed for EGCG.^14^

STD NMR binding studies identified flavanols, catechins, and procyanidins as the main protein ligands to which the observed biological activity can be ascribed. As demonstrated by UPLC-HR-MS analysis, these are the most abundant polyphenolic components in the *Lavado* cocoa extract.

Notably, cocoa polyphenols were shown to be active in vivo, according to results achieved on the ataxic *C. elegans* model expressing the expanded ATX3Q130 protein in neuronal cells. The administration of the polyphenol-enriched fraction of *Lavado* cocoa extracts increased both the mean lifespan and the motility of the animals affected by the expression of the pathogenic protein.

Moreover, as already reported in our previous work,^15^*Lavado* cocoa extracts exert a very potent antioxidant activity useful to counteract oxidative stress, another characteristic feature of neurodegenerative and poly-Q diseases and thus a possible therapeutic target.^40,42^

Collectively, these findings support the use of nutraceuticals based on cocoa polyphenols for the prevention and treatment of SCA3. Given also the antiamyloidogenic activity already reported against Aβ peptides, our results suggest investigating their potential against other neurodegenerative pathologies. Furthermore, from a chemical point of view, this work highlights that catechins and procyanidins are suitable molecular scaffolds for the development of effective anti-SCA3 drugs.

### 4.1 Study Limitations

The main limitation of our study is the lack of tests on vertebrate animals. Nevertheless, the main aim of our work was to verify the possibility of translating the results we obtained on the inhibition of Aβ aggregation to ATX3 protein, a different model of amyloid protein for which, to date, a significantly lower number of effective inhibitors have been identified. The assessment of this effect required a deep in vitro biochemical and structural characterization, as reported here. Nevertheless, a preliminary in vivo assessment of the effectiveness of our natural extracts has been obtained by means of a model organism, *C. elegans*, a useful screening model, especially for toxicity and efficacy studies of chronic treatments. This model also conforms to the 3Rs requirements.

## Supporting Information Available

Detailed UPLC-HR-MS spectrometric data of identified components in *Lavado* cocoa extract and its related polyphenol-enriched fraction (PDF)
