# Supplementary material for: Cell-Free and In Vivo Characterization of the Inhibitory Activity of Lavado Cocoa Flavanols on the Amyloid Protein Ataxin-3: Toward New Approaches against Spinocerebellar Ataxia Type 3
Source: ACS Chem Neurosci. 2023 Dec 28;15(2):278–89. doi: 10.1021/acschemneuro.3c00560 (PMC10797631; doi:10.1021/acschemneuro.3c00560)
Supplement: Supplementary file 1 — cn3c00560_si_001.pdf [file cn3c00560_si_001.pdf]

Supporting Information to:

Cell-free and *in vivo* characterization of the inhibitory activity of *Lavado* cocoa flavanols on the amyloid protein ataxin-3: towards new approaches against spinocerebellar ataxia type 3

Barbara Sciandrone,[#1] Alessandro Palmioli,[#1,2] Carlotta Ciaramelli,[1,2] Roberta Pensotti,[1] Laura Colombo,[3] Maria Elena Regonesi,*[1] Cristina Airoldi*[1,2]

[1]*Department of Biotechnology and Biosciences, University of Milano-Bicocca, P.zza della Scienza 2, 20126 Milan, Italy*

[2]*NeuroMI, Milan Center for Neuroscience, University of Milano-Bicocca, 20126 Milano, Italy.*

[3]*Department of Molecular Biochemistry and Pharmacology - Istituto di Ricerche Farmacologiche Mario Negri IRCCS, Via M. Negri 2, 20156 Milano, Italy.*

[#] These authors contributed equally to this work

*Corresponding authors: cristina.airoldi@unimib.it; mariaelena.regonesi@unimib.it

Table of contents

Table S1. Detailed UPLC-HR-MS spectrometric data of identified components in *Lavado* cocoa extract and its related polyphenols-enriched fraction.

Table S1. Detailed UPLC-HR-MS spectrometric data of identified components in *Lavado* cocoa extract and its related polyphenols-enriched fraction.

RT (min)	Average m/z	Metabolite name	Adduct type	Reference m/z	Error (ppm)	Formula	Abs.ce (nm)	Ontology	INCHIKEY
1.80	165.1407	3-methyl xanthine	$[M-H]^-$	165.1405	1.2	$C_6H_6N_4O_2$	273	Xanthines	GMSNIKWWOQHZGF-UHFFFAOYSA-N
2.95	181.0728	Theobromine	$[M+H]^+$	181.0733	-2.8	$C_7H_8N_4O_2$	273	Xanthines	YAPQBXQYLJRXSA-UHFFFAOYSA-N
3.11	153.0191	Protocatechuic acid	$[M-H]^-$	153.0188	1.9	$C_7H_6O_4$	260, 292	Hydroxybenzoic acid derivatives	YQUVCSBJEUQKSH-UHFFFAOYSA-N
3.45	429.2098	UNKN	$[M-H]^-$	-	-	-	-	-	-
3.68	203.0817	Tryptophan	$[M-H]^-$	203.0826	-4.4	$C_{11}H_{12}N_2O_2$	279, 287	Indolyl carboxylic acids and derivatives	QIVBCDIJIAJPQS-VIFPVBQESA-N
3.91	467.1191	Epicatechin 5-O-beta-D-xylopyranoside	$[M+FA-H]^-$	467.1184	1.5	$C_{20}H_{22}O_{10}$	280, 320	Flavonoid *O*-glycosides	CQVHJPRJVZDENE-VDNHADMDNA-N
4.13	577.1348	Procyanidin B dimer	$[M-H]^-$	577.1359	-1.9	$C_{30}H_{26}O_{11}$	290, 320	Biflavonoids and polyflavonoids	XFZJEEAOWLFHDH-UKWJTHFESA-N
4.18	294.0614	Caffeoyl Aspartate	$[M-H]^-$	294.0619	-1.7	$C_{13}H_{13}NO_7$	280, 314	Aspartic acid and derivatives	YNHFZQQNJPOYRC-DUXPYHPUNA-N
4.38	577.1351	Procyanidin B dimer	$[M-H]^-$	577.1359	-1.4	$C_{30}H_{26}O_{12}$	290, 320	Biflavonoids and polyflavonoids	XFZJEEAOWLFHDH-UKWJTHFESA-N
4.51	289.0721	Epicatechin	$[M-H]^-$	289.0718	1.0	$C_{15}H_{14}O_6$	280, 340	Catechins	PFTAWBLQPZVEMU-UKRRQHHQSA-N
4.68	577.1359	Procyanidin B dimer	$[M-H]^-$	577.1359	0.0	$C_{30}H_{26}O_{11}$	290, 320	Biflavonoids and polyflavonoids	XFZJEEAOWLFHDH-UKWJTHFESA-N
4.72	195.0881	Caffeine	$[M+H]^+$	195.0877	2.1	$C_8H_{10}N_4O_2$	273	Xanthines	RYYVLZVUVIJVGH-UHFFFAOYSA-N
4.80	278.0667	p-Coumaroyl aspartate	$[M-H]^-$	278.0670	-1.1	$C_{13}H_{13}NO_6$	280, 320	Aspartic acid and derivatives	FKBRNPNAUOXZMQ-ZZXKWVIFNA-N
4.89	577.1345	Procyanidin B dimer	$[M-H]^-$	577.1359	-2.4	$C_{30}H_{26}O_{11}$	290, 320	Biflavonoids and polyflavonoids	XFZJEEAOWLFHDH-UKWJTHFESA-N
5.11	305.0676	Gallocatechin	$[M-H]^-$	305.0667	2.95	$C_{15}H_{14}O_7$	280, 340	Epigallocatechins	XMOCLSLCDHWDHP-DOMZBBRYSA-N
5.23	289.0717	Catechin	$[M-H]^-$	289.0718	-0.3	$C_{15}H_{14}O_6$	280, 340	Catechins	PFTAWBLQPZVEMU-DZGCQCFKSA-N

5.42	865.1970	Procyanidin C1 (trimer)	$[M-H]^-$	865.1985	-1.7	$C_{45}H_{38}O_{18}$	280, 340	Biflavonoids and polyflavonoids	MOJZMWJRUKIQGL-XILRTYJMSA-N
5.74	358.0923	Clovamide (Caffeoyl-L-DOPA)	$[M-H]^-$	358.0932	-2.5	$C_{18}H_{17}NO_7$	280, 320	Tyrosine and derivatives	GPZFXSWMDFBRGS-UTCJRWHENA-N
5.90	577.1352	Procyanidin B dimer	$[M-H]^-$	577.1359	-1.2	$C_{30}H_{26}O_{12}$	290, 320	Biflavonoids and polyflavonoids	XFZJEEAOWLFHDH-UKWJTHFESA-N
6.30	311.1398	Phe-Phe	$[M-H]^-$	311.1401	-1.0	$C_{18}H_{20}N_2O_3$	280	Dipeptides	GKZIWHRNKRBEOH-UHFFFAOYNA-N
6.39	342.0795	(Deoxyclovamide) Caffeoyl Tyrosine	$[M-H]^-$	342.0798	-1.0	$C_{18}H_{17}NO_6$	280, 320	Tyrosine and derivatives	JRXLVUMFJASLDR-XBXARRHUNA-N
6.46	463.0875	Isoquercetin	$[M-H]^-$	463.0882	-1.5	$C_{21}H_{20}O_{12}$	280, 340	Flavonoid-3-O-glycosides	OVSQVDMCBVZWGM-QSOFNFLRSA-N
6.81	433.0778	Avicularin	$[M-H]^-$	433.0776	0.5	$C_{21}H_{20}O_{13}$	280, 340	Flavonoid-3-O-glycosides	BDCDNTVZSILEOY-UXYNSRGZSA-N
7.07	326.1030	p-Coumaroyl Tyrosine	$[M-H]^-$	326.1034	-1.2	$C_{18}H_{17}NO_5$	280, 320	Tyrosine and derivatives	LEEDEKWKJVUWGA-BJMVGYQFNA-N
7.34	631.3499	UNKN	$[M+FA-H]^-$	-	-	-	-	-	-
7.61	746.3723	UNKN	$[M+FA-H]^-$	-	-	-	-	-	-
8.49	236.0964	UNKN	$[M-H]^-$	-	-	-	-	-	-
9.67	252.0731	UNKN	$[M-H]^-$	-	-	-	-	-	-
